# Effect of *Metarhizium anisopliae* IPPM202 Extracellular Proteinases on Midgut of *Locusta migratoria manilensis*

**DOI:** 10.3390/insects16111111

**Published:** 2025-10-31

**Authors:** Lei Huang, Zhenghao Wang, Undarmaa Davaasambuu, Hongmei Li, Mark Richard McNeill, Guangchun Cao, Xiongbing Tu, Changzhong Liu, Zehua Zhang, Guangjun Wang, Jing Chang

**Affiliations:** 1 State Key Laboratory for Biology of Plant Diseases and Insect Pests, Institute of Plant Protection, Chinese Academy of Agricultural Sciences, Beijing 100193, China; huanglei9371@163.com (L.H.);; 2 College of Horticulture and Plant Protection, Inner Mongolia Agricultural University, Hohhot 010018, China; 3 Scientific Observing and Experimental Station of Pests in Xilingol Rangeland, Ministry of Agriculture and Rural Affairs, Xilinhot 026000, China; 4 College of Grassland Sciences, Gansu Agricultural University, Lanzhou 730070, China; 5 School of Agroecology, Mongolian University of Life Sciences, Ulaanbaatar 17024, Mongolia; 6 CABI Joint Laboratory, Ministry of Agriculture and Rural Affairs, Beijing 100193, China; 7 Bioeconomy Science Institute, Tuhiraki, Private Bag 4749, Christchurch 8140, New Zealand

**Keywords:** *Metarhizium anisopliae*, extracellular proteases, protease inhibitor, midgut epithelial cell, immune enzymes, entomopathogenic fungi

## Abstract

**Simple Summary:**

*Metarhizium anisopliae* is a fungus used to control insect pests. This study investigated the role of its four extracellular proteases (Pr1, Pr2, Pr3, Pr4) in infecting the migratory locust (*Locusta migratoria*). By using specific protease inhibitors, we found that Pr1 (subtilisin) and Pr4 (cysteine protease) are critical in enabling the fungus to damage the locust midgut and cause death, while Pr2 and Pr3 play no significant role, providing key targets for enhancing locust control strategies.

**Abstract:**

*Metarhizium anisopliae*, an entomopathogenic fungus, can produce four extracellular proteases, subtilisin (Pr1), trypsin (Pr2), metalloproteases (Pr3), and cysteine proteases (Pr4), which are important for pathogenicity of *M. anisopliae* in target hosts. In order to understand their function in *M. anisopliae* pathogenicity, third-instar nymphs of *Locusta migratoria* were fed with a diet containing either conidia of *M. anisopliae* strain IPPM202 or in combination with one of the four inhibitors (TPCK: tosyl-phenylalanine chloromethyl-ketone, inhibitor of Pr1; EDTA: ethylenediaminetetraacetic acid, inhibitor of Pr3; APMSF: 4-amidinophenyl methanesulfonyl fluoride, inhibitor of Pr2; CI1: cathepsin inhibitor 1, inhibitor of Pr4). The effects on mortality, midgut integrity, and the gut enzymes peroxidase (POD), catalase (CAT), superoxide dismutase (SOD), and phenol oxidase (PO) were examined. The results indicated that exposure to IPPM202/TPCK and IPPM202/CI1 caused decreased mortality to *L. migratoria* with no loss of midgut epithelial cellular integrity. On the other hand, exposure to IPPM202/APMSF or IPPM202/EDTA mixtures resulted in higher mortality similar to PPM202, with severely damaged epithelial gut cells with fragmented microvilli, broken endoplasmic reticulum, and disrupted nucleus membrane. The activity of the protective enzymes POD, SOD, CAT, and PO all increased significantly when *L. migratoria* was treated with IPPM202 only, but decreased when any one of the inhibitors was added. We further concluded that TPCK, a subtilisin (Pr1) inhibitor, and CI1, a cysteine protease (Pr4) inhibitor, played important roles in the pathogenicity of the *M. anisopliae* strain IPPM202. Conversely, trypsin (Pr2) and metalloproteases (Pr3) did not have a role in the given process. We further concluded that trypsin (Pr2) and metalloproteases (Pr3) do not contribute to the fungal infection process, while the subtilisin (Pr1) inhibitor TPCK and cysteine protease (Pr4) inhibitor CI1 play critical roles in the pathogenicity of *Metarhizium anisopliae* strain IPPM202, thus providing a foundation for targeted biocontrol strategies.

## 1. Introduction

*Metarhizium anisopliae* (Metchnikoff) Sorokin (Hypocreales: Clavicipitaceae) is one of the most important entomopathogenic fungi widely used for the biological control of insect pests [1,2,3,4,5,6]. The fungus directly penetrates the cuticle and gut membrane of insect pests, using a set of secreted enzymes [7]. The extracellular proteases of *Metarhizium anisopliae* (*M. anisopliae*) can be classified into four functional categories. As the most extensively studied category, Pr1 is a subtilisin-like serine protease that cleaves the C-terminal of phenylalanine (Phe), tryptophan (Trp), or tyrosine (Tyr) and is inhibited by tosyl-phenylalanine chloromethyl-ketone (TPCK) [8,9]. Its protein family is known to contain 11 members. Pr2 is a trypsin-like serine protease that hydrolyzes casein and albumin, albeit with only a quarter of the catalytic activity of Pr1 [10,11]. It is insensitive to TPCK but is partially inhibited by ethylenediaminetetraacetic acid (EDTA) and fully inhibited by 4-amidinophenyl methanesulfonyl fluoride (APMSF) [8,9,10,11,12,13]. Pr3 is a metalloprotease that exhibits thermolysin-like activity on structural and protein substrates [14]. It is capable of hydrolyzing Pr1-specific substrates and is inhibited by EDTA [15,16]. Research on Pr3 in entomopathogenic fungi is more limited than that on Pr1 and Pr2. Pr4 is a cysteine protease characterized by a low isoelectric point [17]. It is inhibited by cathepsin inhibitor 1 (CI1) and exhibits 51% of the activity of Pr1 (though higher than that of Pr2) [18]. It remains the least-studied category in entomopathogenic fungi. Notably, it is postulated that Pr2, Pr3, and Pr4 may all have multiple members. Collectively, these proteases facilitate the penetration of the host cuticle by *M. anisopliae* hyphae, thereby enabling nutrient acquisition, the suppression of host immune responses, and the degradation of host defense molecules [19].

As a major part of the digestive tract, the insect midgut is the target of different types of pathogenic microorganism [20,21]. When *Helicoverpa armigera* (Hübner) (Lepidoptera: Noctuidae) was fed *Beauveria bassiana* spores, its midgut microvilli fell off from the epithelial cells and the gut barriers dissolved and formed holes [22]. Research has indicated that upon ingestion by insect pests, *M. anisopliae* first penetrates the midgut of the intestine and then enters the hemolymph, although it mainly infects hosts through the cuticle [23]. *Spodoptera littoralis* showed rapid mortality after feeding on the diet containing crude soluble protein extract of *M. anisopliae.* Histopathological examination of its intestine showed that the gut epithelium was completely destroyed after 96 h [24]. Preliminary studies carried out in the laboratory showed that toxicity of *M. anisopliae* to *L. migratoria* by ingestion of baits containing spore was significantly higher than that of contact infection through the cuticle and that *M. anisopliae* infection resulted in damage to the midgut epithelial cells and microvilli [25]. Lou found that the pathological sections of the midgut showed that *M. anisopliae* could destroy the integrity of the midgut of *L. migratoria*. After 2 days of feeding, the peritrophic membrane of the midgut dissolved and disappeared, and the microvilli of the midgut gradually fell off after 3 days.

However, the specific roles of individual extracellular proteases (Pr1, Pr2, Pr3, Pr4) in *M. anisopliae* midgut infection remain undefined. To address this gap, four protease inhibitors TPCK, APMSF, EDTA, and CI1 were mixed with *M. anisopliae* spores, and each mixture was fed separately to *L. migratoria*. The purpose of the study was to investigate the relative importance of subtilisin (Pr1), trypsin (Pr2), metalloproteases (Pr3) and cysteine proteases (Pr4) during *M. anisopliae* infection in the midgut of *L. migratoria*. Epithelial cell integrity and enzymatic activity of peroxidase (POD), catalase (CAT), superoxide dismutase (SOD) and phenoloxidase (PO) in the midgut of *L. migratoria* were monitored to illustrate the role of different extracellular protease during *M. anisopliae* infection and colonization and to further clarify the interaction between the *L. migratoria* immune response and *M. anisopliae.*

## 2. Materials and Methods

### 2.1. Metarhizium anisopliae Strain

*M. anisopliae* isolate IPPM202, with high virulence to *L. migratoria*, was cultivated on Potato Dextrose Agar (PDA) medium containing 1% yeast extract at 25 °C. The conidia were harvested from the dried cultures and further vacuum-dried until water content was reduced to less than 5%. The conidia with 95% viability were then sealed in a glass jar and stored at −20 °C [26].

### 2.2. Insect Specimen

The *L. migratoria* used in this study were obtained from a stock colony maintained in an insectary source. Nymphs of *L. migratoria* were reared from eggs to the third-instar stage in wire-mesh-lined cages (45 cm × 55 cm × 50 cm; width × height × depth) at 28 ± 1 °C in a LD 12:12 h photoperiod, and fed daily with fresh glasshouse-grown wheat seedlings (cv. Chinese Zhongmai 175) supplemented with wheat bran. Cages were equipped with an incandescent light bulb to provide additional heat and allow insects to thermo-regulate.

### 2.3. Bait Preparation and Treatments

Inhibitors TPCK, APMSF, EDTA, and CI1 were obtained from Sigma (Sigma-Aldrich, St Louis, MO, USA). The bait consisted of wheat bran and soybean oil mixed at ratio of 100:5 (*W*/*V*) [27]. One treatment contained *M. anisopliae* (IPPM202) only in bait with a concentration of 2.5 × 10^8^ spores/g bran. Four treatments comprised each of the four inhibitors (TPCK, APMSF, EDTA, and CI1) incorporated into the bait, and another four treatments consisted of *M. anisopliae* (IPPM202) with each of the four inhibitors (Table 1). Meanwhile, the bait-only treatment was provided as the control. The concentration of the *M. anisopliae* spore and inhibitors in different treatments are listed in Table 1.

### 2.4. Metarhizium anisopliae Toxicity Bioassay

Third-instar *L. migratoria* were collected from the main colony and 15 nymphs randomly allocated to perforated plastic containers (16 cm × 30 cm × 11 cm) with glass lids and kept in a separate room at 28 ± 1 °C in a LD (12:12 h) photoperiod. The test locusts were starved for approximately 12 h before 0.3 g bait was added to an uncovered plastic Petri dish (90 mm × 10 mm) and placed in each container.

Test insects were allowed to feed on the bait ad libitum (bait without *Metarhizium*) for 24 h, after which all uneaten bait was removed and fresh wheat seedling was supplied until the end of experiment. Each treatment consisted of five replicates with 15 nymphs, and a total of 750 locusts were used for the full experiment. Cages were checked daily for 10 days, the number of dead larvae was monitored and the corresponding mortality was calculated. The cadaverous were removed to minimize cross contamination.

### 2.5. Determination of Gut Epithelial Structure After Feeding on Treatments

In a separate experiment, 3rd-instar *L. migratoria* was treated using the method described above to investigate potential changes in the epithelial structure in the midgut as well as enzyme activity. Four days after treatment, one individual was removed from each container; thus, there were five locusts from each treatment, providing a total of 50 individuals overall. Those nymphs were then euthanized, after which the midguts were dissected and fixed with 2.5% glutaraldehyde in phosphate buffer (pH7.0) for more than 4 h at 4 °C. They were post-fixed with 1% Osmium tetroxide (OsO_4_) for 1 h; dehydrated by a graded series of ethanol and transferred to absolute acetone for 20 min; then placed in 1:1 (*V*/*V*) mixture of acetone/Epon overnight and finally embedded in Epon 618. Ultrathin sections (100 nm) were prepared using an ultramicrotome (Leica EMUC6, Wetzlar, Germany), stained with uranyl acetate and lead citrate, and examined with a Hitachi-500 electron microscope (Hitachi High-Technologies Corporation, Tokyo, Japan) operated at 80 kV.

### 2.6. Measurement of Enzyme Activity After Feeding on Treatments

To measure *L. migratoria* midgut enzyme activity, four days after feeding on the treatments (the same time for gut epithelial structure observation as described above), one nymph from each container was collected (five from each treatment) and euthanized, after which the midgut of each nymph was dissected and immediately homogenized in 500 μL 0.15 M NaCl (1:10 (*w*/*v*)). Gut homogenate was centrifuged at 4 °C, 10,000× *g* for 10 min and the supernatant was used for enzyme bioassays.

Peroxidase (POD), catalase (CAT), and superoxide dismutase (SOD) activity were measured using commercial kits (peroxidase assay kit, CATalase assay kit, superoxide dismutase assay kit; Nanjing Jiancheng Bioengineering Institute, Nanjing, China). Phenoloxidase (PO) was determined according to the procedure outlined by [28] with some modifications, in which 20 μL enzyme preparation and 180 μL of 10 mmol/L catechol were mixed and reacted at 37 °C. Absorbance was read at 420 nm wavelength for 1 h with measurements taken at 60 sec intervals.

### 2.7. Data Analysis

All statistical analyses were performed using software SPSS version 17.0 (SPSS Inc.; Chicago, IL, USA) and the Tukey (HSD) test was used to compare means.

## 3. Results

### 3.1. Metarhizium Anisopliae Toxicity

The mortality of third-instar locust fed with diets containing each of the inhibitors TPCK, APMSF, EDTA, and CI1, was minimal, ranging from 10 to 30% across the four treatments, and not significantly (*p* > 0.10) different from the negative control (26.6%).

**Figure 1:**
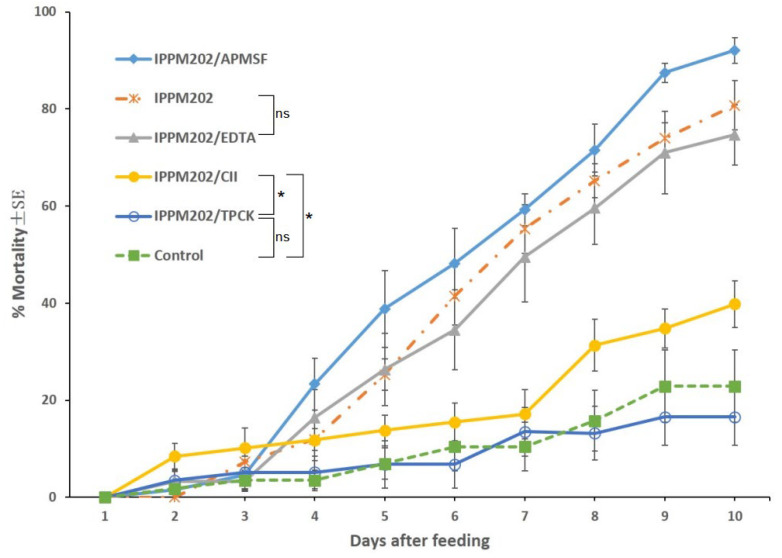
*Locusta migratoria* mortality after ingesting *M. anisopliae* conidia mixed with one of the four selected inhibitors or the control only. Statistical significance: Asterisks (*) indicate statistically significant differences compared to the control group (* *p* < 0.05), and “ns” indicates no significant difference (*p* ≥ 0.05).

*L. migratoria* nymphs feeding on *M. anisopliae* mixed with APMSF (IPPM202/APMSF) had the highest mean mortality of about 90% at the conclusion of the bioassay on the 10th day. Mortality for locusts feeding on *M. anisopliae* mixed with EDTA (IPPM202/EDTA) averaged about 67%, at the conclusion of the bioassay, and not significantly different from that of locusts treated with *M. anisopliae*-only bait (IPPM202, 74%), as given in Figure 1. By comparison, the mortality of locusts feeding on *M. anisopliae* mixed with either CI1 (IPPM202/CI1) or TPCK (IPPM202/TPCK) was 16.0% and 0%, respectively. Mortality on IPPM202/CI1 was significantly different (*p* < 0.05) from both of the IPPM202/TPCK and negative control, while there was no significant difference between mortality for nymphs feeding on IPPM202/TPCK and the negative control.

### 3.2. Gut Epithelial Structure After Feeding on Treatments

**Figure 2:**
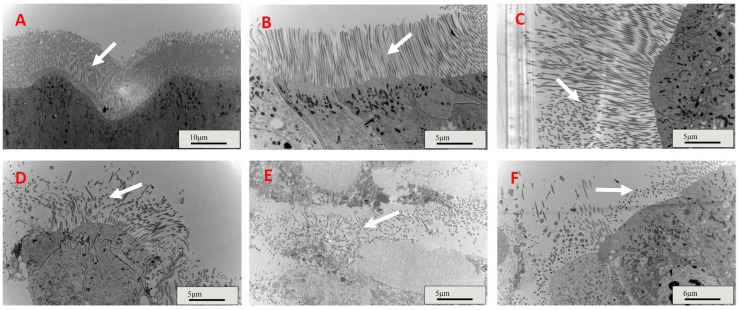
Midgut microvilli structures of *Locusta migratoria* four days after ingestion of *M. anisopliae* whereas. (**A**) Control: only provides bait; (**B**) IPPM202/TPCK: IPPM202 (2.5 × 10^8^ spore/g bran) and *M.anisopliae* protease inhibitor Pr1 (3.52 μg/g bran) were added to bait; (**C**) IPPM202/CI1: IPPM202 (2.5 × 10^8^ spore/g bran) and *M.anisopliae* protease inhibitor Pr4 (19.02 μg/g bran) were added to bait; (**D**) IPPM202: IPPM202 (2.5 × 10^8^ spore/g bran) was added to bait; (**E**) IPPM202/APMSF: IPPM202 (2.5 × 10^8^ spore/g bran) and *M.anisopliae* protease inhibitor Pr2 (2.53 μg/g bran) were added to bait; (**F**) IPPM202/EDTA: IPPM202 (2.5 × 10^8^ spore/g bran) and *M.anisopliae* protease inhibitor Pr3 (146.13 μg/g bran) were added to bait. Note: white arrows represent the microvilli structure under different treatments.

Four days after feeding, the gut microvilli of the control and IPPM202/TPCK-fed nymphs were intact (Figure 2A,B), and that of IPPM202/CI1 fed insects were partially damaged with some fragmented microvilli observed (Figure 2C). However, the gut microvilli of *L. migratoria* fed with IPPM202, IPPM202/APMSF, and IPPM202/EDTA, respectively, exhibited significant breakdown in structural integrity, with the gut epithelial cells swollen and extensive shedding of microvilli observed (Figure 2D–F).

**Figure 3:**
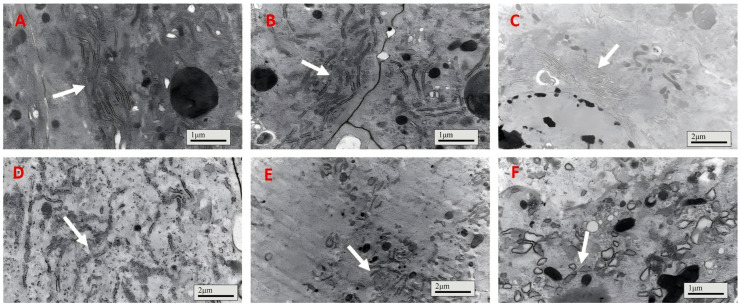
The endoplasmic reticulum of *Locusta migratoria* midgut four days after *M. anisopliae* ingestion, where (**A**) control; (**B**) IPPM202/TPCK; (**C**) IPPM202/CI1; (**D**) IPPM202; (**E**) IPPM202/APMSF; (**F**) IPPM202/EDTA. Note: white arrows the endoplasmic reticulum under different treatments.

The endoplasmic reticulum of the gut epithelial cells in the control group (bait-only) and the IPPM202/TPCK treatment group exhibited good morphology and a well-organized structure (Figure 3A,B). It is worth noting that the endoplasmic reticulum of the gut epithelial cells in the locusts from the IPPM202/CI1 treatment group showed partial fragmentation (Figure 3C), while in the IPPM202/APMSF treatment group, the endoplasmic reticulum underwent evident fragmentation with relatively short fragments (Figure 3E). After treatment with IPPM202, the endoplasmic reticulum was irregularly arranged with indistinct tubular structures (Figure 3D). The most pronounced disruption to the gut epithelial structure was observed in the locusts from the IPPM202/EDTA treatment group, where the endoplasmic reticulum of the gut epithelial cells underwent extensive fragmentation, and the appearance of small vesicles has also been observed (Figure 3F).

**Figure 4:**
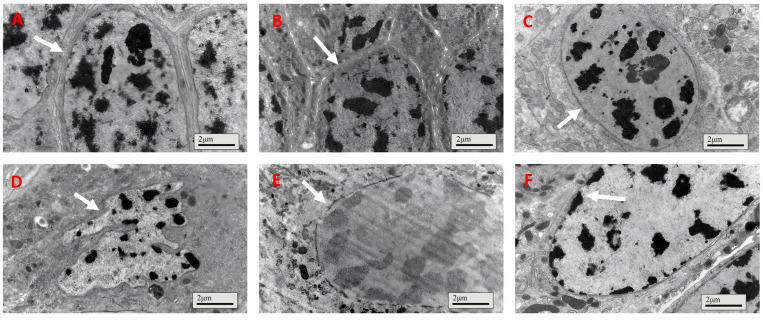
Cell membranes of locust midgut membrane cells four days after ingestion of *M. anisopliae*: (**A**) control; (**B**) IPPM202/TPCK; (**C**) IPPM202/CI1; (**D**) IPPM202; (**E**) IPPM202/APMSF; (**F**) IPPM202/EDTA. Note: white arrows represent the nucleus membrane under different treatments.

For locusts fed the control treatment, the gut cell had a healthy integrated nucleus membrane with dark chromatin inside (Figure 4A); meanwhile, the gut cell nucleus membrane of insects fed IPPM202/TPCK also maintained integrity (Figure 4B). However, the cell nucleus became deformed, nucleus membrane boundaries faded away, and chromatin distribution was smeared after exposure to IPPM202/APMSF, IPPM202/CI1, and IPPM202/EDTA mixtures (Figure 4C,E,F). The most significant disruption to the gut epithelia structure was observed in the insect fed IPPM202, in which the cell nucleus membrane broke down and chromatin was released into the cell plasma (Figure 4D).

### 3.3. Enzyme Activity After Feeding on Treatments

**Figure 5:**
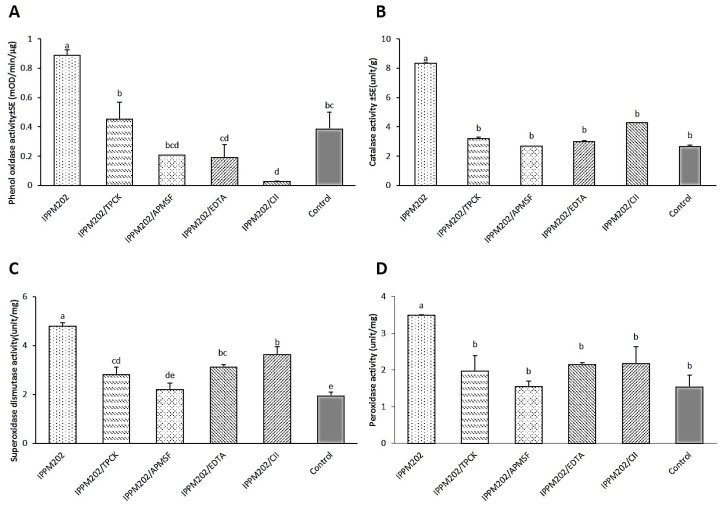
Protective enzyme activity of phenoloxidase (PO), catalase (CAT), superoxide dismutase (SOD), and peroxidase (POD) in the *Locusta migratoria* midgut four days after *M. anisopliae* ingestion. (**A**) Phenoloxidase (PO) activity. (**B**) Catalase (CAT) activity. (**C**) Superoxide dismutase (SOD) activity. (**D**) Peroxidase (POD) activity. Different letters indicate significant differences, while the same letter indicates no significant difference. Lowercase letters represent a significance level of α = 0.05.

The activity of POD, CAT, SOD, and PO enzymes in the gut of *L. migratoria* were all significantly up-regulated after ingestion of IPPM202 compared to the negative control (Figure 5A–D, Table S1). Compared with the treatment of IPPM202 alone, the application of different inhibitors reduced the activity of all detected enzymes to some extent, but the effects varied among inhibitors. Except for PO activity, when a locust was treated with IPPM202/APMSF, IPPM202/EDTA, and IPPM202/CI1, its value was even lower than that of the control. As for each of four inhibitor-only treatments, the activity of POD, CAT, SOD, and PO enzymes from TPCK-treated midgut, compared to the control, all increased by various degrees. Whereas, from those treated with only EDTA or CI1, the activities recorded were decreased for all of the enzymes. The only exception was for APMSF treatment, where POD and SOD activity increased, while CAT and PO activity decreased.

## 4. Discussion

Overall results revealed that locust mean mortality was very high for the application of IPPM202, IPPM202/EDTA, and IPPM202/APMSF, while IPPM202/TPCK and IPPM202/CI1 had low mortality comparable to the negative control. Changes in histology of the midgut epithelial cells of *L. migratoria* in response to *M. anisopliae* varied according to the protease inhibitors added to the bait and reflected the overall mortality. When TPCK and CI1 was mixed with *M. anisopliae* (IPPM202), toxicity declined significantly. Both TPCK and CI1 significantly suppressed the virulence of *M. anisopliae* IPPM202 of 3rd-instar *L. migratoria*. As stated earlier, TPCK is a subtilisin Pr1 inhibitor and CI1 is a cysteine protease Pr4 inhibitor. Thus, we concluded that TPCK and CI1 might affect the virulence of *M. anisopliae* to *L. migratoria* by suppressing its extracellular protease enzyme activity, and subtilisin Pr1 and cysteine proteases Pr4 were the main virulence factors for the *M. anisopliae* IPPM202 isolate. Inhibiting Pr1 or Pr4 absolutely suppressed the virulence of *M. anisopliae*, which shows that there maybe an interaction (or synergy) between Pr1 and Pr4.

Previous studies have found that trypsin Pr2 was not sensitive to TPCK (Pr1′ inhibitor), but was partially inhibited by EDTA (Pr3′ inhibitor) and fully inhibited by APMSF [8,13]. Therefore, EDTA may have a synergetic inhibition interaction between Pr3 and Pr2. However, APMSF and EDTA did not show any interference with the virulence of *M. anisopliae* IPPM202, which implied Pr2 and Pr3 may not be essential in *M. anisopliae* IPPM202 pathogenesis.

Regarding host immune responses, when insects are infected by microorganisms, protective enzymes such as superoxide dismutase (SOD), catalase (CAT) and peroxidase (POD) can be induced to protect cell from toxins produced by damage [29,30]. Meanwhile, humoral immune defense mechanisms are also triggered to defend microorganism attacking through phenol oxidase (PO)-involved melanization [31,32]. In this study, the activities of POD, SOD, CAT, and PO in the host midgut were induced when locusts were fed *M. anisopliae*, and all of these enzymes showed up-regulation in response to the presence of the fungus. However, activity was suppressed for all of four enzymes when different inhibitors were added and compared to *M. anisopliae.* In particular, CAT and POD were not significantly different compared to the control, while the PO and SOD response showed significant variation depending on treatment, and PO activity appeared to be extremely suppressed when locusts had fed on IPPM202/CI1. We inferred that inhibitors used in different treatments may have suppressed the host immune response caused by *M. ansopliae*. Moreover, when the locusts were treated with IPP202/APMSF, the mortality was comparatively higher than the positive control IPPM202, indicating that APMSF may inhibit protective enzyme activity in the locust midgut. Importantly, when locusts were treated independently with different inhibitors, enzyme activity exhibited different degrees of up- or down-regulation compared with the control, but the survival rate of locusts was not affected. Thus, there was no relationship between the observed survival rate of locusts exposed to different treatments and changes in enzyme activity in midgut tissues of cohorts taken for histological examination.

At the same time, we also observed midgut damage—including microvilli destruction and cytoplasmic vacuolation—during the study, indicating a virulence mechanism similar to that of *Bacillus thuringiensis* [33]. Research has suggested that after ingestion of *Bacillus thuringiensis* Cry protoxin by the target insect, the insecticidal crystal protein was activated by digestive enzymes in the midgut and bound to specific receptors in the microvilli of the apical membranes of the columnar cells in the gut [34]. This binding led to spore formation followed by osmotic imbalance between the intracellular and extracellular membranes resulting in cellular disruption. This process destroys the microvilli and the integrity of the gut cell, after which the insect stops feeding and eventually dies [35,36,37,38,39]. Similarly, extracellular proteases secreted by *M. anisopliae* disrupt midgut integrity through similar pathological processes: microvilli destruction, which in turn induces cytoplasmic vacuolation and ultimately cell disruption. Given the critical role of Pr1/Pr4 proteases in mediating this damage, we propose that *M. anisopliae* virulence follows a pathway convergent with B. thuringiensis. Consequently, these results motivate investigating the binding affinity between *M. anisopliae* extracellular proteases (particularly Pr1/Pr4) and insect gut membranes to develop biobased pesticides for *L. migratoria* control.

## 5. Conclusions

This study identifies subtilisin Pr1 and cysteine protease Pr4 as important synergistic factors in *Metarhizium anisopliae* IPPM202 during infection of *Locusta migratoria*. Inhibiting Pr1 (TPCK) or Pr4 (CI1) significantly weakened pathogenicity, reducing mortality to control levels and preserving midgut integrity, whereas Pr2/Pr3 inhibitors (APMSF/EDTA) showed no effect. Infection caused severe midgut damage, driving mortality independently of immune responses. These findings provide new target sites for biocontrol, supporting protease-enhanced fungal strains or receptor-targeting biopesticides for sustainable locust management.

## Figures and Tables

**Table 1:** Concentrations of conidia of *M. anisopliae* (strain IPPM202) and inhibitors used in the *Locusta migratoria* bioassay.

Treatments	Inhibited Proteases of *M. anisopliae*	Conidia Concentration in Diet (Spores/g Bran)	Inhibitor Concentration (μg/g Bran)
IPPM202		2.5 × 10^8^	0
IPPM202/TPCK	Pr1	2.5 × 10^8^	3.52
IPPM202/APMSF	Pr2	2.5 × 10^8^	2.53
IPPM202/EDTA	Pr3	2.5 × 10^8^	146.13
IPPM202/CI1	Pr4	2.5 × 10^8^	19.02
TPCK		0	3.52
APMSF		0	2.53
EDTA		0	146.13
CI1		0	19.02
Control (Bait-only)		0	0

Treatment abbreviations: IPPM202: *M. anisopliae* strain IPPM202 only; IPPM202/TPCK: IPPM202 + TPCK (Pr1 inhibitor); IPPM202/APMSF: IPPM202 + APMSF (Pr2 inhibitor); IPPM202/EDTA: IPPM202 + EDTA (Pr3 inhibitor); IPPM202/CI1: IPPM202 + CI1 (Pr4 inhibitor); TPCK/APMSF/EDTA/CI1: inhibitor-only controls; control (bait-only): negative control (no conidia or inhibitors). Statistical significance: All analyses considered significant at *p *< 0.05 (ANOVA with Tukey’s HSD).

## Data Availability

The original contributions presented in this study are included in the article/Supplementary Materials. Further inquiries can be directed to the corresponding authors.

## Supplementary Materials

The following supporting information can be downloaded at: https://www.mdpi.com/article/10.3390/insects16111111/s1, Table S1: Mean enzyme activity values (±SE) of POD, SOD CAT, and PO measured in the midgut of *L. migratoria* four days following ingestion of treated baits.
