# Supplementary material for: Effect of Metarhizium anisopliae IPPM202 Extracellular Proteinases on Midgut of Locusta migratoria manilensis
Source: Insects. 2025 Oct 31;16(11):1111. doi: 10.3390/insects16111111 (PMC12653306; doi:10.3390/insects16111111)
Supplement: Supplementary file 1 [file insects-16-01111-s001.zip › insects-3785575-supplementary.pdf]

Table S1. Mean enzyme activity values (±SE) of POD, SOD CAT and PO measured in the midgut of *L. migratoria* four days following ingestion of treated baits.

Treatment	POD	SOD	CAT	PO
IPPM202	2.276±0.017a	2.485±0.116a	3.155±0.420a	2.300±0.178a
TPCK	2.081±1.044ab	2.207±0.785ab	1.537±0.473b	2.308±2.285a
APMSF	1.373±0.135bc	1.669±0.128bcd	0.747±0.004cd	0.271±0.051b
EDTA	0.813±0.060c	0.980±0.093ef	0.831±0.261cd	0.452±0.369b
CI1	0.721±0.069c	0.636±0.447f	0.525±0.093d	0.177±0.158b
IPPM202/TPCK	1.286±0.463c	1.450±0.277cde	1.211±0.188bc	1.175±0.522ab
IPPM202/APMSF	1.010±0.158c	1.137±0.232def	1.023±0.047bcd	0.533±0.002b
IPPM202/EDTA	1.396±0.068bc	1.616±0.091bcd	1.121±0.491bcd	0.492±0.396b
IPPM202/CI1	1.417±0.513bc	1.879±0.285bc	1.627±0.552b	0.068±0.012c
Control (Bait–only)	1.000±0.372c	1.000±0.144ef	1.000±0.274bcd	1.000±0.521ab

Key: *M. aniusoplaie stain* IPPM202, Tosyl-phenylalanine chloromethyl-ketone (TPCK), 4-Amidino Phenyl Methane Sulfonyl Fluoride (APMSF), Ethylene Diamine Tetraacetic Acid (EDTA), cathepsin Inhibitor 1 (CI1), peroxidase (POD), superoxide dismutase (SOD), catalase (CAT) and phenoloxidase (PO). Different low case letters above columns indicate statistical differences at $P < 0.05$.
